# Sero-epidemiology of *Coxiella burnetii* in livestock and humans in Isiolo county Kenya

**DOI:** 10.1371/journal.pntd.0013557

**Published:** 2025-10-17

**Authors:** Wilfred Mutuku Mutisya, James M. Akoko, Athman Mwatondo, Mathew Muturi, Daniel Nthiwa, Hussein M. Abkallo, Richard Nyamota, Timothy Wachira, Peter Gathura, Bernard Bett

**Affiliations:** 1 Department of Public Health, Pharmacology and Toxicology, Faculty of Veterinary Medicine, University of Nairobi, Nairobi, Kenya; 2 International Livestock Research Institute, Nairobi, Kenya; 3 Zoonotic Disease Unit, Nairobi, Kenya; 4 Department of Medical Microbiology and Immunology, Faculty of Health, University of Nairobi, Nairobi, Kenya; 5 Faculty of Veterinary Medicine, Dahlem School of Biomedical Sciences, Freie Universität Berlin, Berlin, Germany; 6 Department of Biological Sciences, University of Embu, Embu, Kenya; University of Abuja Faculty of Veterinary Medicine, NIGERIA

## Abstract

**Background:**

*Coxiella burnetii*, the causative agent of Q fever, is a globally distributed pathogen with significant zoonotic and economic impacts, particularly in regions where humans and livestock interact closely. Although endemic in many countries, including Kenya, comprehensive epidemiological data on the pathogen are limited. To address this gap, we conducted a linked human and livestock populations study in Garbatulla, Isiolo County to assess seroprevalence and identify potential predictors of *C. burnetii* exposure.

**Methods:**

We used a cross-sectional design with multistage sampling. Blood and serum samples were collected from 2,157 livestock and 683 humans that were recruited from 242 households. Additional data on herd/household and subject characteristics were collected using a structured questionnaire. Indirect enzyme-linked immunosorbent assay (ELISA) was used to test the serum samples for antibodies against *C. burnetii*. Univariable and multivariable analyses identified potential predictors of exposure in both livestock and humans.

**Results:**

The overall seroprevalence of *C. burnetii* was 47.9% (95% CI: 45.7%-50.1%) in livestock and 44.7% (95% CI: 40.9%-48.5%) in humans. In livestock, significant variation in seroprevalence was found by species (p < 0.001). Goats were found to have significantly higher odds of being exposed to *C. burnetii* compared to cattle, sheep and camels. Both weaners and young animals had significantly lower odds of exposure compared to adults. In humans, the odds of *C. burnetii* exposure were lower among females compared to males. Herds seropositivity was also an important predictor of humans exposure to *C. burnetii.*

**Conclusions:**

This study provides evidence of high seroprevalence of *C. burnetii* in both livestock and humans, highlighting the need for active surveillance programs targeting both populations. These programs should focus on identifying active shedding and implementing targeted control measures to mitigate the public health risks associated with *C. burnetii*.

## Author summary

*Coxiella burnetii* is an important zoonotic pathogen in Kenya that causes illness in humans and livestock in many rural pastoral settings. We conducted a linked human-livestock study to investigate the seroprevalence of *C. burnetii.* We also identified risk factors associated with *C. burnetii* seropositivity. Our study found a high seroprevalence in humans and animals and identified significant risk factors associated with exposure. In livestock, the risk factors included goats and being older. In humans, significant risk factors included being female and residing in a household that had seropositive animals. Implementation of interventions such as targeted awareness campaigns about the disease, species-specific interventions, and promoting safe livestock management practices can potentially reduce the pathogen transmission in livestock and spill over to humans. Future studies should examine the role of wildlife in the transmission of *C. burnetii* and the efficacy of the measures that reduce the risk of transmission.

## Introduction

*Coxiella burnetii*, a zoonotic agent that causes Q fever, is prevalent throughout the world, except in New Zealand and Antarctica [1]. It infects many hosts including animals, birds, arthropods, and humans [2,3]. This is due to the ability of the bacterium to withstand adverse environmental conditions, therefore, increasing the likelihood of infecting a wide range of hosts [4]. In Africa, *C. burnetii* is prevalent in domestic animals and people, particularly in rural areas. Cattle and small ruminants remain the principal source of infection to humans [5]. Q fever is categorized as an occupational hazard for people with close contact with animals, such as animal health workers, pastoralists, abattoir personnel, and research personnel [6,7]. For instance, a past study conducted on cattle in the Somali region found high seroprevalence estimates in humans living in pastoral areas [8].

The transmission of infection to humans is primarily through aerosols contaminated by infected animal birth fluids, urine, or feces. Other potential infection routes include consumption of contaminated milk or milk products and arthropod bites [9–11]. The acute form of the disease is characterized by influenza-like illness, hepatitis, headache associated with photophobia, and the development of atypical pneumonia [12,13]. Other secondary symptoms include chest pain, muscle pain, chronic fatigue, and chills [14,15]. Livestock infections primarily result from exposure to infected animals’ birth products during parturition. In addition, urine and feces may contain *C. burnetii*, which may be a potential source of environmental contamination [16]. Further, some ticks are a potential source of infection, as *C. burnetii* has been detected in multiple tick species [17,18]. In animals, *C. burnetii* infections are in apparent but occasionally abortions and infertility may occur [19,20].

In cattle, age, sex, pastoralism, and wildlife interaction are documented as potential factors for exposure to *C. burnetii* [21,22]. Additionally, livestock trade and porous borders have been documented to facilitate the spread of *C. burnetii* in livestock through the introduction of infected animals into naïve herds [20]. In humans, risk factors for exposure to *C. burnetii* have been identified to include animal sex, age, keeping livestock, illiteracy, and involvement in activities such as animal handling and herding [23–26]. A past systematic review of *C*. *burnetii* epidemiology across Africa documented evidence of its endemicity among livestock and humans. That review documented seroprevalence estimates ranging from 7% to 33% in sheep, 4% to 55% in cattle, and 1% to 32% in humans [5].

In Kenya, livestock serological estimates of 18–40% in goats, 13–20% in sheep, 0–28% in cattle, and 5–46% in camels have been reported [27–29] with seroprevalence varying with geographical locations, husbandry practices, and production systems among other factors.

Despite *C. burnetii* being an important pathogen due to its high endemicity there is limited epidemiological data in both animals and humans [5,30]. As a result, *C. burnetii* has not attracted adequate attention in animal and public health surveillance systems [31]. This is exacerbated by misdiagnosis and under-reporting in health facilities [32]. Additionally, the lack of sufficient knowledge on the risk factors of the disease among the local communities, veterinarians, medical professionals, and public health workers as well as the absence of a coordinated approach in surveillance and control further contributes to the spread of *C. burnetii* worldwide [6,20].

While studies in separate host species are important and informative, a multidisciplinary approach toward understanding the dynamics of *C. burnetii* transmission and key intervention areas is necessary for its control [33]. We conducted a linked livestock-human survey to evaluate individual and household or herd-level associations and the potential predictors that contribute to exposure among the target population. The findings of this study have provided evidence on the extent of seroprevalence of *C. burnetii* in the study population, the associated risk factors and thus serve as a basis for developing informed strategies to prevent and control the pathogen in the area.

## Materials and methods

### Ethics statement

We received ethical clearance for the study from the Institutional Research Ethics Committee at the International Livestock Research Institute (reference number ILRI-IREC2020-07). Livestock owners consented to livestock sampling after receiving adequate information on the study. Further, the individuals participating did so voluntarily, following full briefing on the study scope and objectives. Written consents were provided for adults (18 years and above); for children aged between 13–17 years, written assent forms and parental/guardian permissions were obtained. For children under 13 years, only written parental/guardian permissions were obtained.

### Study area

**Fig 1:**
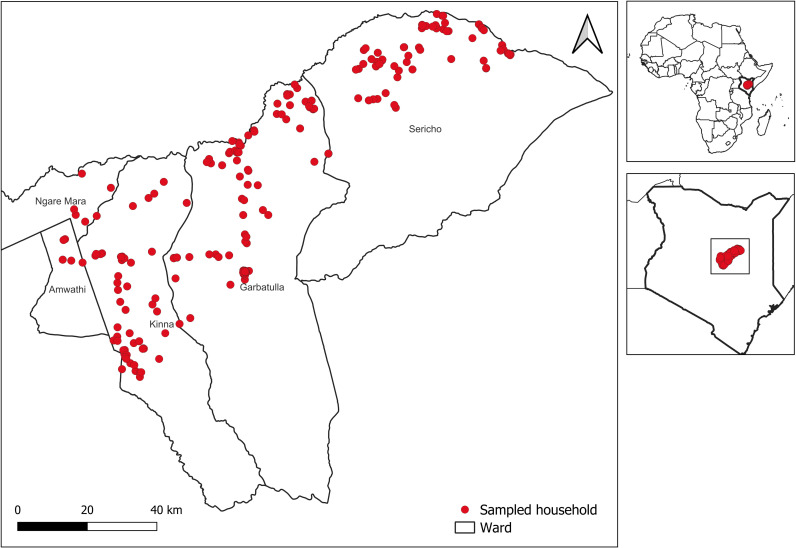
A map of Africa and Kenya highlighting the sampling locations within the Garbatulla sub-county. The datasets for the wards and Kenya boundary were freely obtained from DIVA-GIS (diva-gis.org/data.html), and those for African countries were downloaded from the Open Africa website (https://open.africa/dataset/?tags=Africa).

This study was conducted in the Kinna, Garbatulla, and Sericho wards of Garbatulla Sub-County, Isiolo County, northern Kenya (Fig 1) between July and August 2021. The study area was previously described in recent publications [34,35]. These wards lie within an arid to semi-arid agro-ecological zone, where pastoralism, subsistence agriculture, and small-scale trade are the primary sources of livelihood [36]. The region is characterized by low altitude, with Kinna at the highest elevation of 589 meters above sea level and Sericho at the lowest, at 459 meters above sea level. The climate is predominantly hot and dry throughout the year, with a mean annual temperature of 29°C. The area receives an average annual rainfall of 580 mm, with November and April being the wettest months [37]. The three wards were chosen for the study due to their proximity to Meru National Park, Bisanadi Game Reserve, and two other wildlife sanctuaries. This unique location facilitates significant livestock-wildlife-human interactions, which could enhance the potential for zoonotic disease transmission.

### Sample size determination

The sample size for humans and livestock was calculated using a standard formula for estimating a population proportion [38]. An assumed *C. burnetii* seroprevalence of 50% was used, with a precision level *(d)* of 0.05 and a *Z*-value of 1.96 for 95% confidence. This yielded a baseline sample size of 384 for both humans and animals. To account for clustering at the herd and household levels, the sample size was adjusted using the formula *DE = 1 + *ρ*(m − 1)*, where *m* is the expected number of samples per herd or household and **ρ*(rho)* represented the intra-cluster correlation coefficient (ICC), which was set at 0.2, consistent with prior studies [39]. It was assumed that up to 20 animals per herd and at least three humans per household would be sampled [40]. After adjusting for clustering, the final sample sizes were 1,843 animals and 538 humans.

### Sampling design

The multistage random sampling technique was used to identify sampling locations and human and livestock subjects for the study. The sampling locations were identified using 250 random geographical coordinates (RGCs) generated with QGIS version 3.4.4 (https://qgis.org). Households within 0.5 kilometers of an RGC were eligible if they had at least one adult who consented and any of the four livestock species (cattle, camels, sheep, or goats). In cases where coordinates fell near a homestead (a shared enclosure housing animals from multiple households), one household that had the four species of interest, and a consenting adult person was randomly selected using simple random sampling method. A household was defined as a group of individuals sharing a common living space, including a cooking area. At the same time, a herd was described as a group of livestock from a selected household containing any of the four species of interest.

The systematic random sampling technique was used to select up to five healthy animals per species from each herd. Herds with fewer than five animals were fully sampled. Sick animals and those under one month old were excluded. For humans, all consenting household members who owned the selected herds were included, except children under two years of age.

Sampling was conducted by certified veterinarians and nurses/clinicians for animals and humans, respectively. Up to 10 mL of venous blood was collected from each animal from the jugular vein, while in humans, about 5 mL of venous blood was obtained from the median cubital vein. Samples were collected in barcoded vacutainer tubes, transported in cool boxes (4–8°C) to a field laboratory, and processed. Blood samples were centrifuged at 2,500 rpm for 15 minutes to extract sera, which were aliquoted into 2 mL barcoded cryovials. The sera were then transported at -20°C in motorized cooler boxes to the International Livestock Research Institute laboratory in Nairobi for *C. burnetii* antibody screening.

### Data collection

After animal and human sampling, structured questionnaires were administered using Open Data Kit (ODK) software to collect epidemiological data on potential predictors of *C. burnetii* exposure. Data on livestock included species kept, herd size, age, and sex. Additional information was gathered on wildlife interactions and inter-herd breeding practices within households. The questionnaire collected details on age, gender, occupation, and education level for humans. Other factors recorded included handling animal fetal membranes and consumption of unpasteurized milk.

### Laboratory testing

Livestock sera were screened for antibodies against *C. burnetii* using the ID Screen Q Fever Indirect Multi-species Kit (IDvet, Montpellier, France), while human serum samples were tested using the SERION ELISA Classic *C. burnetii* Phase II IgG Kit (Virion/Serion, Germany). Both assays were performed according to the manufacturer’s instructions, with all samples and assay controls tested in duplicates. Optical densities (ODs) were recorded using a Synergy BioTek microplate ELISA reader (Synergy, BioTek, Winooski, VT, USA), at wavelengths of 450 nm for the ID Screen Q Fever kit and 405 nm for the Serion ELISA kit. The ODs from each test kit were interpreted following the manufacturer’s instructions. For sera that were borderline (suspect), re-testing was performed, and those that yielded the same result as initially were considered negative for data analysis.

### Statistical analysis

The data were analyzed using R statistical software (version 4.2.2) [41]. Field and laboratory data were cleaned, merged, and stored in a single comma-delimited (.csv) file for analysis. Descriptive statistics were generated using contingency tables created with the *gmodels* package [42]. These statistics included estimates of the total seroprevalence of the pathogen in each host category, stratified by various categorical explanatory variables. To calculate 95% confidence intervals (CIs) for the seroprevalence estimates, we used the *epiR* package [43]. In livestock, explanatory variables of interest included species, sex, age, herd size, and ward (Kinna, Garbatulla, and Sericho). For humans, seroprevalence was stratified by demographic characteristics (age, sex, occupation, and education level), history of febrile illness, history of miscarriage, consumption of unpasteurized milk, and occupational practices such as handling animal fetal membranes. Associations between categorical variables and *C. burnetii* exposure were initially evaluated using the chi-square test.

Univariable and multivariable analyses were conducted using generalized linear mixed-effects models (GLMMs) implemented using the *glmer* function in the *lme4* package [44]. Household ID was included as a random effects variable to adjust for within-household correlations. A causal web model was developed to guide the selection of variables for risk factor analysis. This approach prioritized proximal predictors in the causal pathway while excluding intervening or endogenous variables.

For livestock, independent predictors included age, sex, species, and herd size. Variables such as ward, climate, and dominant soil type were treated as correlated; dummy variables for the ward were included to account for clustering. History of abortion and infertility were excluded as these were considered outcomes of *C. burnetii* infection. For humans, key exposure factors included consumption of unpasteurized milk and handling animal fetal membranes, while demographic variables (age, sex, education level, and occupation) were included as covariates to generate adjusted measures of effect.

Independent variables with p-values < 0.20 in the univariable analysis were selected to construct the initial multivariable mixed-effects models for both human and livestock data. Model selection was guided by Akaike’s Information Criterion (AIC), with the model having the lowest AIC value chosen as the best fit. Two-way interaction terms between independent variables were included to test for interaction effects in the final multivariable models.

A likelihood ratio test (LRT) was used to assess the statistical significance of both the fixed and random effects in all the models fitted to the data [45]. Additionally, the clustering of *C. burnetii* exposure within herds and households was addressed by calculating the intra-class correlation coefficient (ICC) and variance estimates of the final multivariable models using the *sjstats* package [46].

## Results

### Descriptive results

A total of 2,157 livestock were sampled from 231 herds. Goats constituted the largest proportion of the sampled animals (44.2%, n = 953), followed by sheep (39.4%, n = 849), cattle (12.0%, n = 258), and camels (4.5%, n = 97). The distribution of sampled animals across the wards varied, with Garbatulla contributing the highest proportion (45.3%, n = 977), followed by Kinna (29.2%, n = 629) and Sericho (25.5%, n = 551). Regarding sex distribution, more females were sampled (77.0%, n = 1,660) compared to males (23.0%, n = 497).

In total, 683 human individuals from 242 households were sampled. The majority were males (83.0%, n = 567), with females making up 17.0% (n = 116). A significant portion of the sampled individuals had no formal education (76.1%, n = 520), followed by those with primary education (17.1%, n = 117) and post-primary education (6.7%, n = 46). The primary source of livelihood among participants was livestock keeping (86.2%, n = 589), followed by housewives (7.2%, n = 49), students (3.2%, n = 22), employed individuals (2.6%, n = 18), and livestock traders (0.7%, n = 5). Age-wise, the largest group was 21–40 years (39.3%, n = 269), followed by those aged 41–60 years (26.0%, n = 177), 4–20 years (22.7%, n = 155), and those over 60 years (12.0%, n = 82). The median age of the subjects was 34 years and the range of 4–82.

### Seroprevalence of *C. burnetii* in livestock

The overall seroprevalence of *C. burnetii* in livestock was 47.9% (95% CI: 45.7%–50.1%). Nearly all herds (99.9%) had at least one seropositive animal. Seroprevalence varied significantly by species (*χ²* = 335.3, df = 3, *p* < 0.001), with goats showing the highest seroprevalence, followed by sheep, camels, and cattle in that order (Table 1). Age significantly influenced seroprevalence (*χ²* = 103.8, df = 2, *p* < 0.001), with adult animals recording the highest seroprevalence, followed by young animals, and weaners in that order. Geographically, seroprevalence was highest in Garbatulla, followed by Kinna and Sericho in descending order (Table 1).

**Table 1:** Seroprevalence of *C. burnetii* in livestock stratified by categorical independent factors.

*Variable*	*Category*	*N*	*% seroprevalence (95% CI)*	*Odds ratio (95% CI)*	*p-value*
*Species*	Goat	953	66.6 (63.6-69.7)	1.0 (Ref)	
	Sheep	849	41.5 (38.0-44.9)	0.3 (0.3-0.4)	<0.001
	Cattle	258	7.0 (4.3-10.0)	0.0 (0.0-0.1)	<0.001
	Camel	97	28.9 (20.6-38.3)	0.2 (0.1-0.3)	<0.001
*Sex*	Female	1660	48.3 (46.0-51.0)	1.0 (Ref)	
	Male	497	46.1 (41.6-50.7)	0.9 (0.7-1.1)	0.297
*Age*	Adults	1793	52.6 (50.2-55.0)	1.0 (Ref)	
	Weaners	292	2.8 (0.9-4.7)	0.3 (0.2-0.4)	0.001
	Young animals	72	8.3 (4.2-15.2)	0.1 (0.0-0.2)	<0.001
*Ward*	Garbatulla	977	51.8 (48.6-55.2)	1.0 (Ref)	
	Kinna	629	48.2 (44.2-52.3)	0.8 (0.6-1.1)	0.179
	Sericho	551	40.7 (36.5-44.9)	0.6 (0.4-0.8)	0.001

*Ref, reference category; CI, confidence interval.*

### Seroprevalence of *C. burnetii* in humans

Overall, 80.8% of households had at least one seropositive human subject. Male individuals had a higher seroprevalence compared to females. Seroprevalence of *C. burnetii* did not differ significantly by age groups, and Wards (Table 2).

**Table 2:** Seroprevalence of *C. burnetii* in humans stratified by categorical factors.

*Variable*	*Category*	*N*	*% seroprevalence (95% CI)*	*Odds ratio (95% CI)*	*p-Value*
*Age*	0-20 years	155	41.3 (33.5-49.2)	1.0 (Ref)	
	21-40 years	269	42.8 (36.8-48.9)	1.1 (0.7-1.7)	0.651
	41-60 years	177	47.7 (42.2-57.4)	1.5 (0.9-2.3)	0.386
	>60 years	82	46.3 (36.6-58.2)	1.2 (0.7-2.2)	0.47
*Gender*	Male	567	47.4 (43.2-51.7)	1.0 (Ref)	
	Female	116	31.0 (23.3-40.0)	0.5 (0.3-0.8)	0.002
*Ward*	Garbatulla	267	45.3 (39.3-51.6)	1.0(Ref)	
	Sericho	238	42.9 (36.6-49.4)	0.9 (0.6-1.3)	0.471
	Kinna	178	46.1 (38.8-53.7)	1.1 (0.7-1.6)	0.835

*Ref, reference category; CI, confidence interval.*

### Univariable analysis of risk factors for *C. burnetii* exposure in livestock and humans

The univariable analyses identified animal age, species, wards, and herd size as the significant risk factors for *C. burnetii* exposure in livestock, as shown in Table 3.

**Table 3:** Risk Factors for *C. burnetii* Exposure in Livestock Identified Using Univariable Mixed-Effects Logistic Regression Models.

Variable	Category	N	% seroprevalence (95% CI)	Odds ratio (95% CI)	p-value
*Animal age*	Adults	1793	52.6 (50.2-55.0)	1.0 (Ref.)	
	Weaners	292	2.8 (0.9-4.7)	0.3 (0.2-0.4)	0.001
	Young animals	72	8.3 (4.2-15.2)	0.1 (0.0-0.2)	0.0001
*Sex*	Female	1660	48.3 (46.0-51.0)	1.0 (Ref.)	
	Male	497	46.1 (41.6-50.7)	0.9 (0.7-1.1)	0.266
*Species*	Goat	953	66.6 (63.6-69.7)	1.0 (Ref.)	
	Sheep	849	41.5 (38.0-44.9)	0.3 (0.3-0.4)	0.016
	Cattle	258	7.0 (4.3-10.0)	0.0 (0.0-0.1)	<0.001
	Camel	97	28.9 (20.6-38.3)	0.2 (0.1-0.4)	<0.0001
*Ward*	Garbatulla	977	51.8 (48.6-55.2)	1.0 (Ref.)	
	Kinna	629	48.2 (44.2-52.3)	0.8 (0.6-1.1)	0.179
	Sericho	551	40.7 (36.5-44.9)	0.6 (0.4-0.8)	0.001
*Herd size*	1-200	1248	45.6 (42.8-48.6)	1.0 (Ref.)	
	>200	910	51.0 (47.7-54.5)	1.2 (1.0-1.4)	0.111

*Ref: reference category; CI: confidence interval.*

The analysis revealed that females had significantly lower odds of exposure, while individuals with no formal education, farmers, employed persons, those who consumed unpasteurized milk, and individuals with seropositive animals in their herds had higher odds of *C. burnetii* exposure compared to those who did not participate in such practices. A significant association was found between human exposure and herd-level exposure, with 92.8% of exposed individuals coming from households with seropositive herds (χ² = 5.9, df = 1, p = 0.021) (Table 4).

**Table 4:** Risk Factors for *C. burnetii* Exposure in Humans Identified Using Univariable Mixed-Effects Logistic Regression Models.

Variable	Category	N.	(% seroprevalence (95% CI)	Odds ratio (95% CI)	p-Value
*Gender*	Male	567	47.4 (43.2-51.7)	1.0 (Ref.)	
	Female	116	31.0 (23.3-40.0)	0.5 (0.3-0.8)	0.002
*Age*	0-20 years	155	41.3 (33.5-49.2)	1.0 (Ref.)	
	21–40 years	269	42.8 (36.8-48.9)	1.1 (0.7-1.7)	0.651
	41–60 years	177	47.7 (42.2-57.4)	1.5 (0.9-2.3)	0.386
	>60 yrs.	82	46.3 (36.6-58.2)	1.2 (0.7-2.2)	0.47
*Education*	Post-secondary	46	37.0 (23.9-51.0)	1.0 (Ref.)	
	Primary	117	36.8 (28.2-45.7)	1.1 (0.5-2.1)	0.981
	No education	520	47.1 (42.7-51.6)	1.6 (0.8-3.0)	0.183
*Occupation*	Student	22	27.3 (13.6-47.6)	1.0 (Ref.)	
	Farmer	589	48.4 (44.3-52.7)	2.6 (1.0-7.1)	0.058
	Housewife	49	22.5 (12.2-34.1)	0.8 (0.2-2.7)	0.719
	Livestock trader	5	40.0 (20.0-91.2)	2.1 (0.3-17.7)	0.491
	Employed	18	55.6 (0.0-14.9)	0.1 (0.0-1.4)	0.096
*Consumption of unpasteurized milk*	No	33	30.2 (18.6-44.8)	1.0 (Ref.)	
	Yes	640	45.6 (41.7-49.7)	2.0 (1.0-4.1)	0.057
*Handling animal fetal membranes*	No	649	44.7 (40.8-48.8)	1.0 (Ref.)	
	Yes	33	42.4 (27.3-60.0)	0.9 (0.4-1.9)	0.728
*Herd seroprevalence*	No	378	87.0 (83.9-90.3)	1.0 (Ref.)	
	Yes	305	92.8 (90.2-95.5)	2.0 (1.1-7.3)	0.021

*Ref; reference category; CI, confidence interval.*

### Multivariable Analysis of risk factors for *C. burnetii* Exposure in Livestock

The multivariable analyses on livestock data suggested that animal age, and species were significant risk factors (p < 0.05) for *C. burnetii* exposure, as shown in Table 5.

**Table 5:** Risk Factors for *C. burnetii* Exposure in Livestock Identified Using Multivariable Mixed-Effects Logistic Regression Models.

Variable	Category	Odds ratio (95% CI)	Coefficient	Z	SE	p-value
*Animal age*	Adults	1.0 (Ref.)				
	Weaners	0.4 (0.3-0.5)	-1.029	-6.442	0.160	<0.0001
	Young animals	0.1 (0.0-0.3)	-2.208	-4.653	0.475	<0.0001
*Animal Species*	Goat	1.0 (Ref.)				
	Cattle	0.0 (0.0-0.1)	-3.195	-11.602	0.275	<0.0001
	Sheep	0.3 (0.3-0.4)	-1.373	-11.166	0.105	<0.0001
	Camel	0.3 (0.1-0.5)	-1.343	-4.208	0.319	<0.0001
*Ward*	Garbatulla	1.0 (Ref.)				
	Sericho	0.8 (0.6-1.1)	-0.174	-1.121	0.155	0.262
	Kinna	1.2 (0.9-1.6)	0.195	1.344	0.145	0.179

*Ref; reference category; CI, confidence intervals; SE, standard error; Log likelihood ratio test =* *-1255.50, number of observations = 2157, number of households = 231; Random effect variable (household id) variance = 0.26(95% CI: 0.23-0.28).*

### Multivariable analysis of risk factors for *C. burnetii* Exposure in Humans

The final multivariable analyses revealed that interaction with seropositive herds and being a female were identified as significant independent predictors (p < 0.05) for *C. burnetii* exposure in humans, as summarized in Table 6.

**Table 6:** Risk Factors for *C. burnetii* Exposure in Humans Identified Using Multivariable Mixed-Effects Logistic Regression Models.

Variable	Category	Odds ratio (95% CI)	Coefficient	SE	Z	p-value
*Gender*	Male	1.0 (Ref.)				
	Female	0.5 (0.3-0.7)	-0.774	0.228	-3.391	0.001
*Herd seroprevalence*	No	1.0 (Ref)				
	Yes	2.2 (1.3-4.0)	0.807	0.297	2.714	0.007

*Ref; reference category; CI, confidence intervals; SE, standard error; Log likelihood ratio test =* *-459.1, number of observations = 683, number of households = 242; Random effect variable (household id) variance = 0.19 (95% CI: 0.00-0.80).*

## Discussion

We investigated the epidemiology of *C. burnetii* infection using a linked study that involved livestock and humans from randomly selected households in Garbatulla sub-county, Isiolo. The study area is pastoral rangeland with a large diversity of livestock and wildlife that share grazing and watering resources. This setting provides ideal conditions for the transmission of multi-host infectious agents. Our study detected high seroprevalences of *C. burnetii* in humans and livestock. The estimated seroprevalence in humans was comparable to those that have been reported in Egypt (of 10–32%) [47] and among the Fulani ethnic group in Togo (45.5%) [12]. It was however higher than those that have been reported in Marsabit (13.2%), Tana River/Garissa (24.4%), and Kajiado (26.0%) counties in Kenya [40,48,49]. It was also higher than the mean seroprevalence (<8%) that was reported in a systematic review of *C. burnetii* in Africa [5].

Our study found no significant difference in seroprevalence among individuals of different age groups. However, higher seroprevalence estimates were detected in individuals aged 40–60 years and above compared to younger people. This finding corresponds to those of past similar studies [40,50] and could be attributed to the cumulative risk of exposure in older people. We also recorded higher seroprevalence in males compared to females. This finding corresponds to that of a previous study in similar settings [40] which found significantly higher seroprevalence estimates in male individuals compared to females. This could be attributed to the different roles played by men compared to women in rural pastoral settings. Lastly, there was no significant difference in seroprevalence among individuals from the sampled wards although high seroprevalence estimates were recorded in individuals based at Kinna compared to those in Garbatulla. This could be attributed to the observed mass human-livestock migration from both Sericho and Garbatulla wards in search of better pastures thus increasing the risk of exposure.

Most of the observed *C. burnetii* seroprevalences in livestock were within the expected past documented ranges. Previous studies in Kenya and Ethiopia under similar settings, for example, reported *C. burnetii* seroprevalence in goats of 83.1% and 54.2%, respectively [49,51]. The seroprevalence estimate that we obtained in cattle (7.0%) was lower than that in camels (28.9%), a result that was consistent with a previous similar study that was conducted in Marsabit [48]. The estimate we observed in sheep (41.5%) was higher than the 6–20% range reported in a similar study in Kenya [30]. The seroprevalence of *C. burnetii* was high in goats, sheep, and camels, these livestock species thrive well in pastoral areas, compared to cattle. Small ruminants are generally recognized as being the primary reservoirs of the pathogen [52] this could be attributed to the higher seroprevalence observed in goats, sheep, and camels which are often reared together.

High seroprevalence estimates were recorded in adult animals compared to weaners and young animals. This finding corresponds with previous studies [40,53,54] since older animals just like humans might have had repeated exposure in their lifetime. In addition, high seroprevalence estimates were recorded in female animals compared to males. This finding corresponds with that of a past similar study [40]. This could be attributed to the close contact of female animals with birth fluids and fetal membranes which are known to contain high numbers of the pathogen. In addition, females are known to stay longer in farms compared to males thus increasing their risk of exposure. Livestock in the Garbatulla ward had the highest seroprevalence estimates among the three wards we sampled. This finding could be attributed to the geographical location of Garbatulla in between Sericho and Kinna. The proximity to Sericho, which is fairly arid could increase the risk of exposure through airborne dispersal. In addition, Garbatulla is commonly used as a migration route to Kinna for both humans and livestock in search of better pastures.

Lastly, we observed high seroprevalence estimates in livestock sampled from larger herd sizes (>200) compared to smaller ones a finding that corresponds to similar past studies [54,55] which suggests a positive correlation between herd size and *C. burnetii* seroprevalence. This could be attributed to the high levels of contact in livestock in large herd sizes which favors transmission of the pathogen.

Our multivariable analyses identified risk factors for *C. burnetii* exposure in both humans and animals. They also detected a significant clustering of *C. burnetii* exposure in households/herds. In humans, being female was associated with lower odds of exposure while the presence of seropositive animals in herds raised within the household was associated with increased odds of exposure to the pathogen. This finding concurs with those of the past similar studies [12,20,40,56,57] which found that males have a greater risk of exposure compared to females. It is documented that men are often more involved in activities such as herding, assisting with animal deliveries, working in abattoirs, and milking, which increase their exposure due to more frequent interactions with infected animals compared to women [58]. This highlights the complexity of gendered exposure to zoonotic infections and suggests that seroprevalence studies must account for nuanced and context-dependent differences in gendered livelihood practices.

The observed positive association between herd-level *C. burnetii* seroprevalence and human exposure contrasted with reports in other similar studies on zoonotic diseases [20,40]. For instance, in one of the studies [40], 21.6% of individuals who tested positive for antibodies against *C. burnetii* did not have any seropositive animals in their herd. This greatly differs from our finding that 92.8% of exposed individuals came from households with seropositive herds. This variation could be attributed to the varied transmission routes for *C. burnetii* within different geographical locations and suggests that other unobserved factors might have been involved. Moreover, the seroprevalence of *C. burnetii* in livestock was significantly associated with age, with both young animals and weaners showing considerably lower odds of exposure compared to adult animals. This finding, corresponds to those from previous studies [40,59,60] and could be possibly attributed to the to the cumulative repeated exposure in adult animals as documented by past similar studies [28,61]. This suggests that exposure dynamics might vary depending on specific ecological, environmental, or management factors. In addition, it underscores the need to target younger animals in control programs, such as vaccination or biosecurity measures, which traditionally focus more on mature animals. Further, our study identified goats as having higher odds of exposure compared to the other livestock species. A finding that corresponds to those of past similar studies [28,40]. This could be explained by the fact that goats have been shown to play an important role in the transmission of *C. burnetii* to humans [59].

Livestock-wildlife interactions are also prevalent in pastoral areas, and these may play a pivotal role in the epidemiology of this and other zoonotic agents [28,62]. However, wildlife was excluded from this study, limiting our understanding of their role in the spread of this pathogen in the region.

The study had several limitations. First, it was conducted during the dry season, when herds tend to converge due to the scarcity of feed resources. The combination of this herd convergence, dusty conditions, and overstocking during such periods may explain the high exposure rates observed in both humans and livestock. Additionally, the study design employed has limitations, as it relies on data collected at a single point in time without repeated sampling of the study population [63]. To obtain a more accurate assessment of current infections and their sources, future studies should incorporate designs and methods that allow for repeated sampling [64]. Finally, previous research [65,66] has highlighted the potential for serological cross-reactions between *C. burnetii* and other pathogens such as *Bartonella* spp. and *Chlamydia* spp. Given these findings, it is possible that the seroprevalence in our study population was overestimated due to such cross-reactions, and hence the variations with past similar studies conducted in the country [33,49,67–72].

## Conclusion

Overall, these findings underscore the interconnectedness of human and animal health in pastoralist settings, where cultural practices and herd management play central roles in pathogen transmission. Interventions aimed at reducing *C. burnetii* exposure among these communities should consider the specific risk factors identified in both livestock and human populations, particularly those related to gender roles and animal interactions. Enhanced awareness, targeted public health interventions, and preventive measures focusing on safe animal management practices could significantly mitigate the transmission of *C. burnetii* and reduce its public health impact among pastoralist populations.

### Recommendations

To address the high seroprevalence of *C. burnetii* in pastoralist communities, targeted awareness campaigns should be developed to educate both men and women about the risks associated with livestock exposure. These campaigns should emphasize the heightened exposure risk in goats, as well as the age-related immunity differences observed in livestock. Public health messaging should be adapted to local cultural practices and risk factors, ensuring that both genders understand the potential for exposure and the steps they can take to reduce their risk.

Given the high seroprevalence of *C. burnetii* in goats, sheep and camels, species-specific intervention strategies should be prioritized. Efforts should focus on improving veterinary care and potentially vaccinating these species of livestock to reduce the transmission of *C. burnetii*. Additionally, it is important to investigate the geographic variability in seroprevalence to understand the role of local ecological and management factors in driving pathogen transmission. By targeting areas with higher exposure risks, more tailored and effective interventions can be implemented.

In light of the findings that females exhibited significantly lower odds of seropositivity (protective) compared to males, gender-specific health programs should be developed. These programs should address the potential differences in immune responses, behaviors, and environmental exposures that may influence men’s susceptibility to *C. burnetii*. To address the close interspecies transmission dynamics highlighted by the study, interventions should focus on reducing human-animal interactions. Given the positive association between human and herd seroprevalence, efforts should be made to minimize exposure to infected livestock by improving herd management practices. This includes promoting safe handling procedures, using personal protective equipment (PPE), and limiting the movement of potentially infected animals. Reducing direct contact between humans and infected herds is essential in curbing the spread of *C. burnetii*.

In addition, the promotion of safe animal management practices should be a priority in areas with high exposure risks. Training programs should be introduced to improve hygiene and animal care, educating pastoralists about best practices to control the spread of *C. burnetii*. These practices could include improved sanitation, proper disposal of animal waste, and measures to limit the spread of the pathogen through animal and human contact.

To better understand and control the spread of *C. burnetii*, enhanced surveillance and monitoring systems are needed. These systems should track seroprevalence in both human and animal populations to detect outbreaks early and assess the effectiveness of intervention measures. Continuous monitoring will ensure that appropriate actions are taken to mitigate the transmission of the pathogen, especially in high-risk regions.

To address the study limitations, future studies should aim to include multiple seasons to account for potential seasonal variations in animal and human exposure to *C. burnetii*. This would provide a more comprehensive understanding of how environmental factors, such as herd density and overstocking during dry periods, contribute to the spread of the pathogen. Additionally, efforts to investigate the role of herd management practices during different seasons, including grazing and movement patterns, could further elucidate their impact on exposure rates.

Regarding the study design, incorporating repeated sampling in future research would allow for a more accurate assessment of *C. burnetii* infections and their temporal dynamics. Longitudinal studies would provide valuable insights into infection trends over time and help to better understand the sources of exposure. Furthermore, to mitigate the potential overestimation of seroprevalence due to cross-reactions with other pathogens like *Bartonella spp.* and *Chlamydia spp.*, future studies should include confirmatory diagnostic methods, such as PCR-based testing, to more precisely differentiate between infections caused by *C. burnetii* and those caused by other pathogens. This would enhance the accuracy of the results and provide clearer data on the true prevalence of *C. burnetii* exposure.

Finally, a One Health approach, which integrates human, animal, and environmental health strategies, should be adopted. Collaboration between veterinary, public health, and environmental sectors is essential for developing comprehensive solutions to manage the transmission of *C. burnetii*. By fostering cross-sectoral cooperation, the broader ecological and socio-cultural factors that influence pathogen spread can be addressed in a more coordinated and effective manner.

## Supporting information

S1 Data This Excel file contains anonymized data collected from human participants involved in the study. Variables include demographic information, potential predictors, and serological test results for *Coxiella burnetii*. All personally identifiable data have been removed per ethical and legal requirements.
(XLSX)

S2 Data This Excel file contains anonymized data from livestock associated with the participating households. Variables include herd demographics, potential predictors, and *Coxiella burnetii* serological results. No identifiable farm or owner information is included in the dataset.
(XLSX)

S1 Fig The causal web illustrates the hypothesized relationships between socio-demographic, occupational, and behavioral factors influencing exposure to *Coxiella burnetii* in humans. The yellow nodes represent exposure or risk factors, while the blue node represents the outcome variable. Green arrows indicate the direction of causal influence.
(PNG)

S2 Fig This causal web illustrates the hypothesized relationships between animal-level and herd-level factors influencing exposure to *Coxiella burnetii* in livestock. Yellow nodes represent potential risk factors, while the blue node denotes the outcome variable. Green arrows indicate the direction of causal influence.
(PNG)
