# Supplementary figures and images for: Sero-epidemiology of Coxiella burnetii in livestock and humans in Isiolo county Kenya
Source: PLoS Negl Trop Dis. 2025 Oct 17;19(10):e0013557. doi: 10.1371/journal.pntd.0013557 (PMC12551958; doi:10.1371/journal.pntd.0013557)

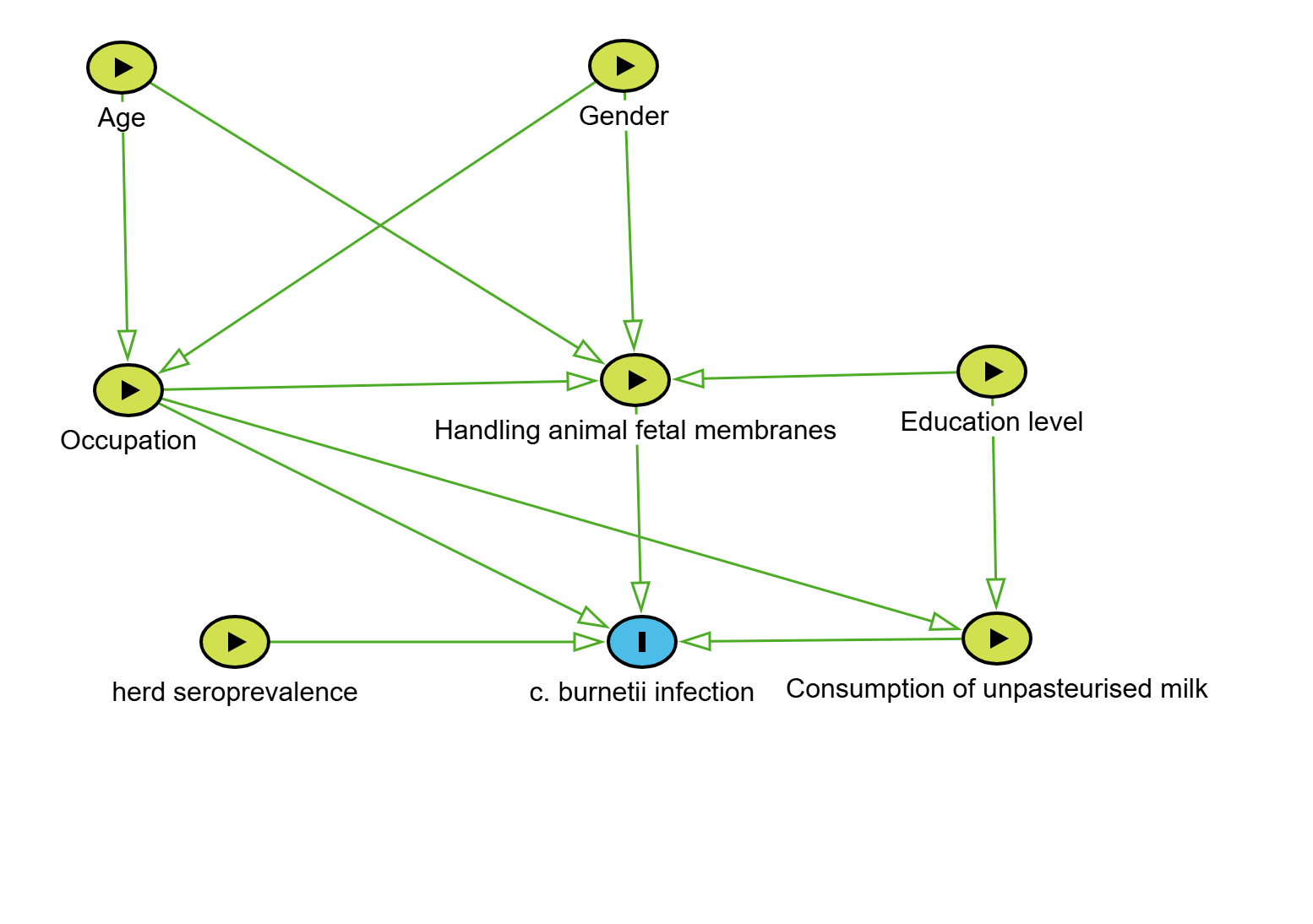

Supplement: S1 Fig — The yellow nodes represent exposure or risk factors, while the blue node represents the outcome variable. Green arrows indicate the direction of causal influence. (PNG) [file pntd.0013557.s003.png]

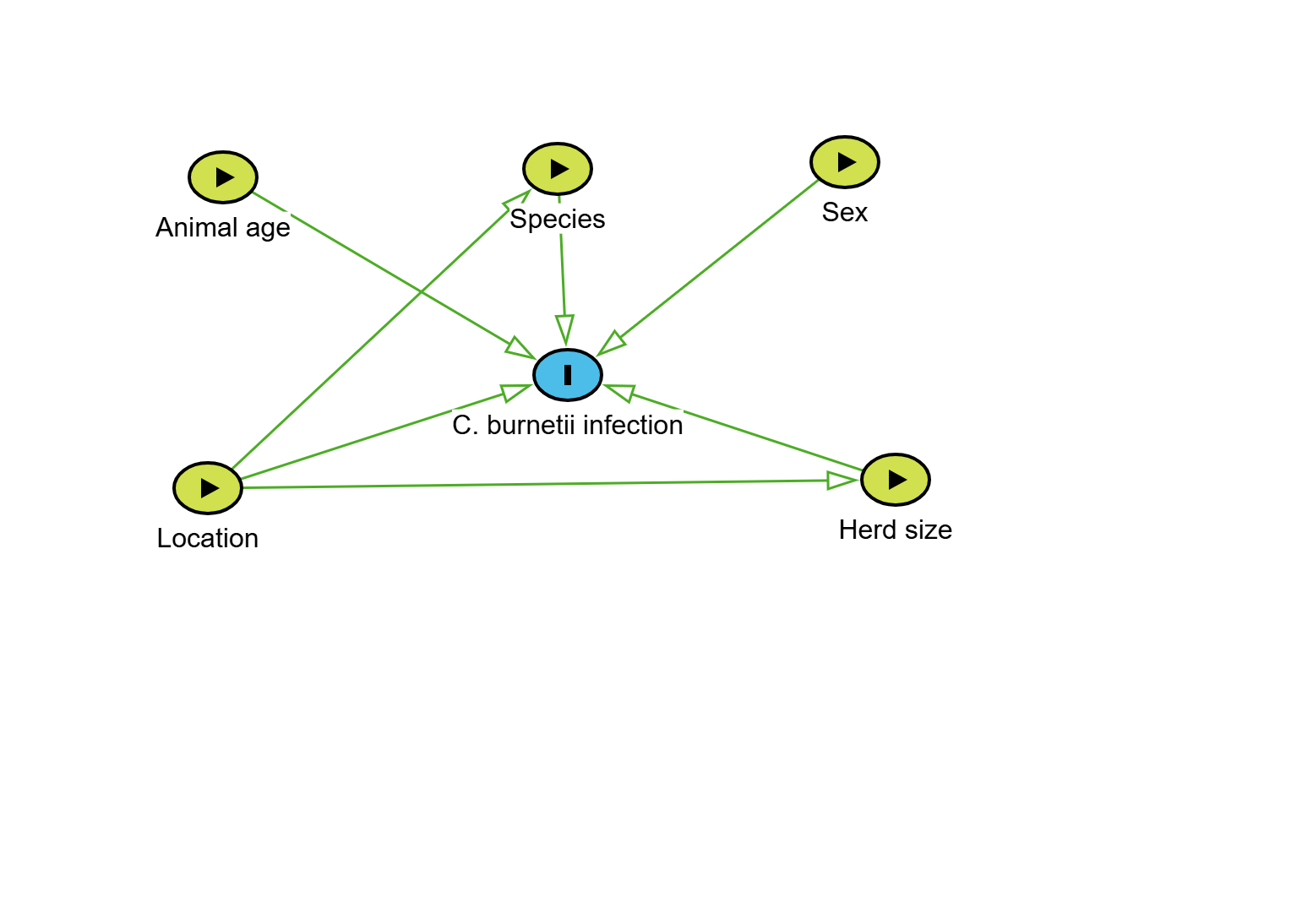

Supplement: S2 Fig — Yellow nodes represent potential risk factors, while the blue node denotes the outcome variable. Green arrows indicate the direction of causal influence. (PNG) [file pntd.0013557.s004.png]
